# Defensive Abdominal Rotation Patterns of Tenebrionid Beetle, *Zophobas atratus*, Pupae

**DOI:** 10.1673/031.012.13301

**Published:** 2012-11-11

**Authors:** Toshio Ichikawa, Tatsuya Nakamura, Yoshifumi Yamawaki

**Affiliations:** ^1^ Department of Biology, Faculty of Sciences, Kyushu University, Fukuoka 812-8581, Japan; ^2^ Basic Life Science, Graduate School of System Life Sciences, Kyushu University, Fukuoka 812-8581, Japan

**Keywords:** appendages, defensive behavior, gin trap, mechanoreceptor, sensilla

## Abstract

Exarate pupae of the beetle *Zophobas atratus* Fab. (Coleoptera: Tenebrionidae) have free appendages (antenna, palp, leg, and elytron) that are highly sensitive to mechanical stimulation. A weak tactile stimulus applied to any appendage initiated a rapid rotation of abdominal segments. High-speed photography revealed that one cycle of defensive abdominal rotation was induced in an all-or-none fashion by bending single or multiple mechanosensory hairs on a leg or prodding the cuticular surface of appendages containing campaniform sensilla. The direction of the abdominal rotation completely depended on the side of stimulation; stimulation of a right appendage induced a right-handed rotation about the anterior-posterior axis of the pupal body and vice versa. The trajectories of the abdominal rotations had an ellipsoidal or pear-shaped pattern. Among the trajectory patterns of the rotations induced by stimulating different appendages, there were occasional significant differences in the horizontal (right-left) component of abdominal rotational movements. Simultaneous stimulation of right and left appendages often induced variable and complex patterns of abdominal movements, suggesting an interaction between sensory signals from different sides. When an abdominal rotation was induced in a freely lying pupa, the rotation usually made the pupa move away from or turn its dorsum toward the source of stimulation with the aid of the caudal processes (urogomphi), which served as a fulcrum for transmitting the power of the abdominal rotation to the movement or turning of the whole body. Pattern generation mechanisms for the abdominal rotation were discussed.

## Introduction

Holometabolous insects account for more than 80% of all known insect species worldwide. They are characterized by a pupal stage in which there are dramatic changes from a larva to adult with respect to anatomy, physiology, behavior, and ecology. This dissociation between the larval and adult stages of these insects may promote their exploitation of a wide variety of environments. Thus, the evolution of the pupal stage in the insect life history seems to be vital for the extraordinary diversity and success of the insect group. The pupal stage is usually a quiescent phase in the life of an insect, wherein pupae may be susceptible to attack by predators and parasitoids. Pupae are usually covered with a hardened cuticle and are often protected within a cocoon or cell (Hinton 1955); in addition, some species of beetles utilize chemical defenses (Daloze et al. 1995; Schröder et al. 1998). Active, behavioral defensive mechanisms are known for the pupae of many Coleoptera and some Lepidoptera species. Pupal appendages, including legs, antennae, palps, and wings, are usually fixed to the body (obtect type) or free from the body (exarate type). The free appendages of exarate pupae appear to be vulnerable to attack from predators (Ichikawa and Kurauchi 2009).

The abdomen of exarate pupae is often armed with sclerotized margins (i.e., jaws or spines), and active movement of the abdomen can play an important role in defense. Pupae exhibit different patterns of abdominal movements against different sensory stimulations induced by potential enemies. The exarate pupae of the beetle *Tenebrio molitor* exhibit at least two types of abdominal movements: abdominal rotation in response to tactile stimulation of appendages (Hollis 1963; Askew and Kurtz 1974) and closure of sclerotized jaws (gintrap) in response to stimulation of the abdominal intersegmental region (Hinton 1946; Wilson 1971). The former appears to function as an effective defense against the cannibalistic larvae of tenebrionid beetles *T. molitor* and *Zophobas atratus* (Ichikawa and Kurauchi 2009).

Tactile stimuli are received by mechanoreceptive sensors on the cuticular surfaces of insects; these are either hair- or dome-shaped (campaniform) sensilla that are usually specialized to respond to different types of mechanical stimuli, including touch, air currents, sound, vibration, and cuticle deformation (Keil 1997; Iwasaki et al. 1999). The pupae of many species of insects are covered with several cuticular hairs of different shapes and lengths (Hinton 1946; Bouchard and Steiner 2004). These hairs may function as protectors and as mechanical sensors to perceive the external environment. The distribution and function of the mechanoreceptive sensilla on the pupal body surface have been intensively examined in the tenebrionid beetle, *Z. atratus* Fab. (Coleoptera: Tenebrionidae) (Kurauchi et al. 2011). Long hair sensilla (80–200-µm long) are mostly located on the lateral regions of each segment of the body, and short hairs (5— 50-µm long) are distributed on the surface of almost all parts of the pupal body except the wing lobes (elytra) and intersegmental regions of the abdomen. In addition to the hair-shaped sensilla, all appendages, including the elytra and abdominal intersegmental regions, have many campaniform sensilla that can encode strains in the cuticular exoskeleton. A physiological study revealed that the campaniform sensilla may be the major mechanoreceptor type that mediates the two types of defensive behaviors mentioned above (Kurauchi et al. 2011).

To understand the neuronal mechanisms of pupal defensive responses, the spatial and temporal patterns of the responses of *Z. atratus* were analyzed. This is the first report concerning an abdominal rotation response induced by tactile stimulation of appendages; the accompanying paper describes the second gin-trap closure response (Ichikawa et al. 2012).

## Materials and Methods

### Animals and preparations

Giant mealworms, *Z. atratus*, were purchased from a local supplier as completely grown larvae. The detritivorous or omnivorous larvae were maintained under crowded conditions in a mixture of peat moss and sawdust and were fed fresh Japanese radishes (*Raphanus sativus* L. var. *longipinnatus* L.H.Bailey). Individual larvae were isolated in a plastic cup for pupation. The pupae were maintained at 26 ± 1° C under a 16:8 L:D photoperiod for an average pupal period of 13 days.

One-day-old pupae were usually used for the analysis of the abdominal rotation response under restrained or unrestrained conditions. The dorsal part of the thorax of a pupa was fixed to a platform with melted paraffin and maintained under an undisturbed condition at 25–26° C for more than 12 hours before use. For the unrestrained condition, a pupa was laid on its dorsal side or lateral side down on a flat substrate covered with a sheet of sandpaper.

Whether a complete abdominal rotation could be performed in a pupal cell was examined. To make the pupal cell, a larva was released into a plastic bottle stuffed with leaf mold. The shape of the pupal cell was measured by pouring plaster of Paris into the cell. On average, pupal cells had a depressed elliptical floor 53.6 × 43.6 mm and a dome 23.9 mm high (n = 9). A pupa was placed in the center of the floor of an artificial pupal cell without the dome structure.

### High-speed photography

Restrained or unrestrained pupae were illuminated with four cold light sources, and abdominal movements were recorded by one or two digital video cameras (HAS-200R; Ditect, http://www.ditect.co.jp/) with a resolution of 640 × 480 pixels at 200 frames/s. Images were stored on a PC as avi-formatted files through a video-capturing interface. To register the coordinates of body parts, sequential still images were imported into video-tracking software (Dipp-Motion XD; Ditect). The positions of a particular portion of the pupal body (usually tip of the abdomen) were manually plotted frame-by-frame (5-ms interval) to register their two- or threedimensional coordinates on the PC screen. The data were saved into a text file and subsequently pasted into an Excel extraction file for further analysis.

### Mechanical stimulation

Abdominal rotation responses were induced by a tactile stimulation of a mechanosensory hair or cuticular surface of an appendage. A single sensory hair or group of hairs was stimulated manually with a piece of silver wire (0.2 mm in diameter) under a binocular microscope. Since the probability of a response induced by this method was relatively low and variable among different pupae, most responses were induced by applying mechanical forces on a part of cuticle that contained many campaniform sensilla (Kurauchi et al., 2011). This procedure was performed by pressing a fire-polished tip of a curved glass rod (1.2 mm in diameter) attached to an arm of a galvanometer. The arm was moved by injecting a current pulse (50 ms, 1–5 V) into the galvanometer by using an electronic stimulator (Nihon-koden, http://www.nihonkohden.com/). The force was measured with a calibrated strain gauge. The force (1–10 mN) was usually adjusted so that an abdominal rotation response occurred with a probability of > 50%. For the simultaneous stimulation of two appendages, another galvanometer-driven glass rod was added. A preliminary experiment indicated that no significant response habituation occurred when the interval of mechanical stimulation exceeded 30 s. Thus, the tactile stimuli were delivered at 1-minute intervals throughout the experiment. The sites of stimulation on individual appendages were usually the anterior surface of a maxillary palp, lateral surface of the third flagellomere of an antenna, distal portion of the femur (or tarsus) of a leg, and distal portion of the elytron.

### Analysis of the trajectory of abdominal rotations

Since the trajectory of the abdominal motion in a posterior view was usually ellipsoidal, the lengths of the long and short axes of an ellipsoidal pattern on a two-dimensional plane were usually measured for comparison. The velocity of an abdominal rotation was calculated from changes in the threedimensional positions of the tip of the abdomen obtained by analyzing images recorded by two cameras positioned perpendicular to each other.

## Results

### Rotation patterns induced by unilateral stimulation

**Figure 1.**
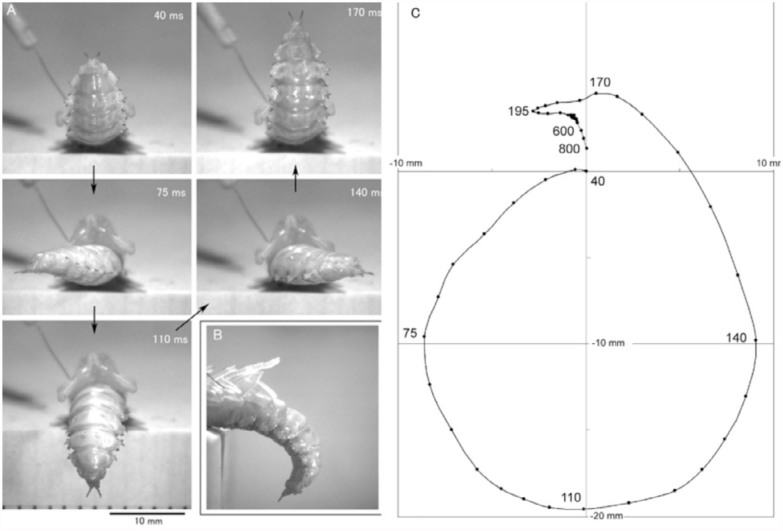
Posterior and lateral views of pupal abdominal rotation induced by tactile stimulation of a single mechanosensory hair on the femur of right middle leg. (A) Photographs show particular phases of the abdominal rotation and their timing after the onset of stimulation. (B) Lateral view of a strong backward bending of the abdomen. (C) Trajectory of the tip of the abdomen during the rotation, plotted at 5-ms intervals. The direction of rotation was anticlockwise in a posterior view. High quality figures are available online.

The cephalic and thoracic appendages of a pupa were very sensitive to mechanical stimulation; a tactile stimulus applied to only one sensory hair could trigger an abdominal rotation in an all-or-none fashion. However, the efficacy of the stimulation of the sensory hair was usually low and ranged from 5–40% among different pupae. Figure 1A shows high-speed photographs of an abdominal rotation induced by the tactile stimulation of a single sensory hair on the right-middle leg. The abdomen started to rotate approximately 40 ms after the onset of stimulation, rotated 90° at 75 ms, 180° at 110 ms, 270° at 140 ms, and 360° at 170 ms. When the abdomen rotated 180°, the abdomen curved more than 90° dorsally (Figure 1B). The trajectory of the tip of the abdomen plotted at 5-ms intervals was ellipsoidal or pear-shaped (Figure 1C). The rapid rotation of the abdomen stopped at 220 ms with a slight overshooting beyond the medial plane of the body. The abdomen subsequently returned very slowly to the original position in 1 s. The velocity of the abdominal rotation was variable; the rotation greatly accelerated during the late (return) phase of rotation and started to decelerate before the abdomen rotated 360°. The latency from the contact of the hair to the first detectable motion of the abdomen in the pupa varied from 35–50 ms. The mean latency (± SD) of the pupa was 42.5 ± 12.5 ms (n = 20). The durations of the rapid rotation of the abdomen (from the start to the stop of rotation) ranged from 170–190 ms. The average mean latency in 20 different pupae was 45.3 ± 10.5 ms. The durations of the rapid abdominal rotation in the 20 pupae ranged from 120–210 ms, and the mean duration was 156 ± 53.3 ms.

**Figure 2.**
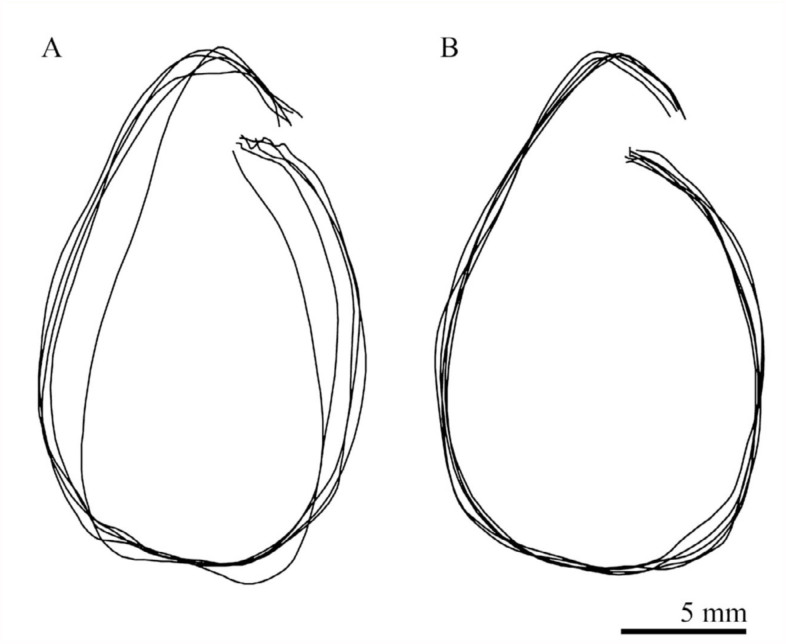
Variability of trajectory patterns of clockwise rotations of the abdomen induced by stimulation of a single (A) and multiple (3) sensory hairs (B) on the left middle leg femur. High quality figures are available online.

The direction of abdominal rotation completely depended on the side of stimulation; stimulation of a right appendage induced a right-handed rotation about the anterior-posterior axis of the pupal body and vice versa. When looking at the pupa lying dorsal side down posteriorly on the platform, the right-handed rotation appeared anticlockwise (Figure 1), whereas the left-handed rotation appeared clockwise. The abdominal rotations induced by stimulation of the same single hair exhibited some variability in the shape of the trajectory of abdominal rotation (Figure 2A). When multiple (2–4) sensory hairs close together were stimulated, the probability of responses often increased as the number of stimulated hairs increased. Furthermore, the variability of the rotation often decreased (Figure 2B).

Similar rotational patterns could be readily induced by tactile stimulation of the cuticular surface of any appendage with the tip of a glass rod. When comparing many abdominal rotations induced by repetitive stimulations of the same single appendage at 1-minute intervals, there was some variability in the amplitude of the horizontal (left-right) component of rotation motions, while the vertical (dorsal-ventral) components of motion were almost constant; the difference in the amplitude was usually in the range of 10–20% for the former and < 2% for the latter. These results suggest that the abdomen always bended to maximum flexion in the vertical direction, but not in the horizontal direction.

**Figure 3.**
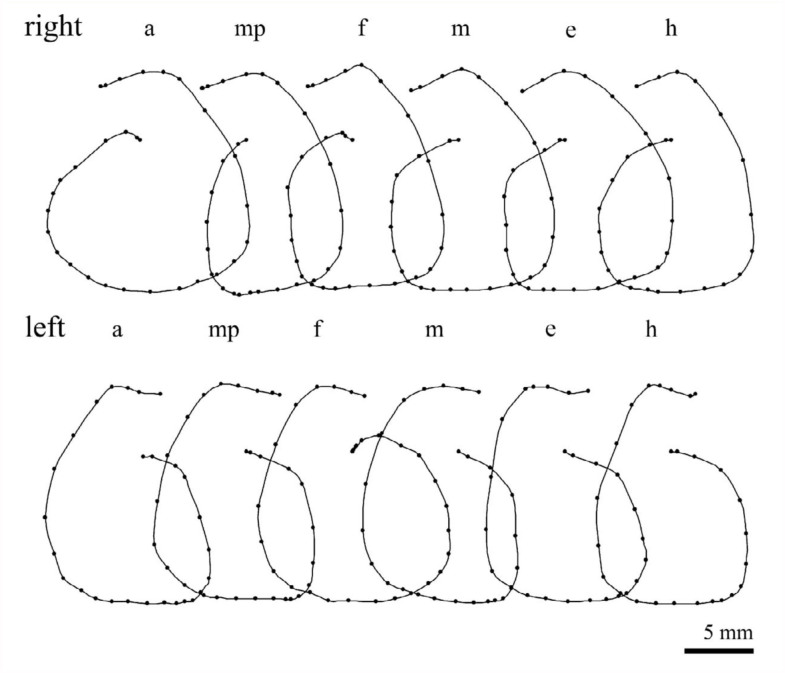
Typical trajectory patterns of abdominal rotations induced by the stimulation of individual appendages on both sides of a pupa, a, antenna; mp, maxillary palp; f, fore leg; m, middle leg; h, hind leg; e, elytron. The horizontal component of abdominal rotation induced by stimulation of the right maxillary palp (mp) appeared to be smaller than that induced by stimulation of thoracic appendages (m, h, and e). High quality figures are available online.

**Figure 4.**
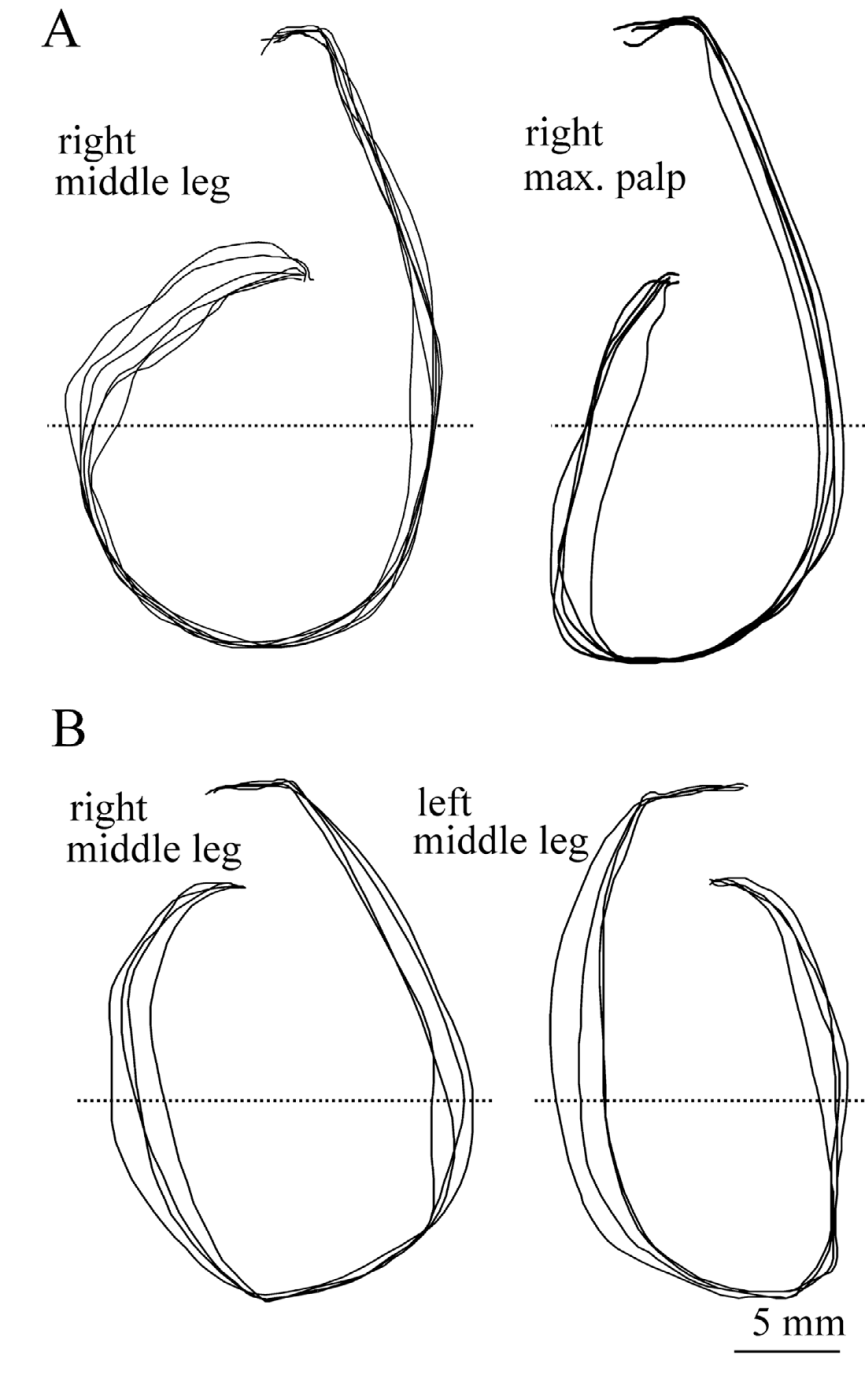
Differences in the trajectory patterns of abdominal rotations induced by the stimulation of different appendages on the same side (A) and stimulation of the same types of appendages on different sides (B). Amplitudes of the horizontal components of abdominal motion measured at the level of dotted line differed significantly between the two appendages (*p* < 0.01, Student's *t*-test). High quality figures are available online.

Stimulation of different appendages also revealed some variability in the horizontal components of rotation as shown in Figure 3. In the pupa, the anticlockwise rotation induced by stimulation of a right maxillary palp was significantly smaller than the other anticlockwise rotations induced by stimulation of thoracic appendages (i.e., legs and elytra). When the amplitudes of the horizontal components of rotations induced by stimulation of a maxillary palp and a middle leg on the same side were analyzed in 20 pupae, the latter was significantly larger than the former in nine of them (Figure 4A). Significant differences in the amplitudes of rotation were often observed between rotations of opposite directions induced by stimulation of the same appendages on different sides (Figure 4B). The side of stimulation inducing a larger rotation of abdomen varied from pupae to pupae.

### Abdominal movement patterns induced by bilateral stimulation

**Figure 5.**
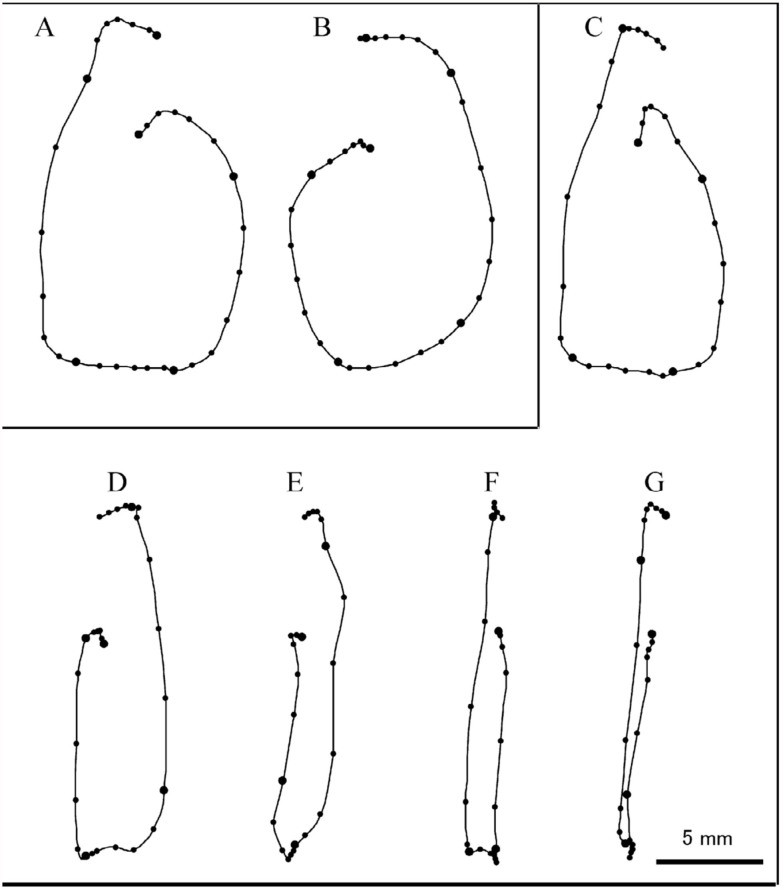
Variability of abdominal rotation (or swing) patterns induced by unilateral (A, B) and bilateral (C-G) stimulations of right and left middle legs. Note the various levels of reduction in the horizontal components of rotation induced by bilateral stimulation. The start of the abdominal rotation or swing was defined as time = 0 when the tip of the abdomen moved > 0.5 mm in the next frame. The positions of the tip were plotted every 5 ms (small dots) or 30 ms (large dots). Latencies between the onset of stimulation and start of rotation (or swing) were 35 (A), 45 (B), 35 (C), 65 (D), 120 (E), 65 (F), and 70 ms (G). High quality figures are available online.

To clarify the interactions and processing mechanisms of sensory signals from mechanoreceptors in the right and left appendages, the two appendages were stimulated simultaneously. The majority of responses induced by bilateral stimulation were simple clockwise or anticlockwise rotations, similar to the rotations induced by unilateral stimulation (Figure 5A, B). However, others showed characteristic trajectory patterns that were compressed along the horizontal axis; they often had a sharp edge indicating an abrupt change in the movement direction (Figure 5C–G). The compressed trajectory patterns were probably due to different degrees of reduction in the horizontal component of abdominal movement. The greatest reduction of the components changed the rotatory motion of the abdomen to a vertical swing of the abdomen (Figure 5G). In addition to the change in the spatial patterns, bilateral stimulation induced a temporal change. It was often observed that the start of abdominal movement was somewhat delayed; the latencies of the abdominal movement in the pupa illustrated in Figure 5 were 35–120 ms (mean ± SEM = 68.6 ± 8.6 ms, n = 11) and 35–55 ms (mean ± SEM = 47.1 ± 3.1 ms, n = 7) in the cases of bilateral and unilateral stimulation, respectively.

### Rotational velocity

**Figure 6.**
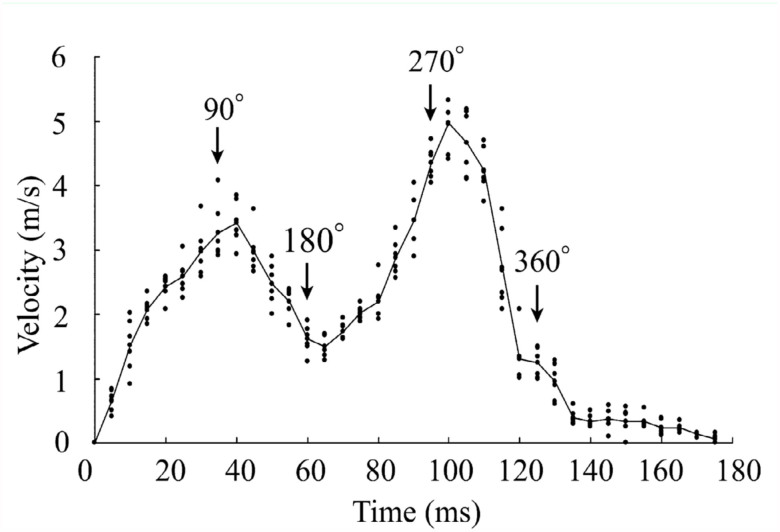
Velocity profiles of abdominal rotations. Averaged values of seven determinations of rotational velocity (dots) calculated every 5 ms in a single pupa are connected by thick lines. The angles 90°, 180°, 270°, and 360° indicate the phases of abdominal rotation. High quality figures are available online.

The rotational velocity was calculated from the three-dimensional trajectory of the tip of the abdomen. Figure 6 shows a typical velocity profile with dual peaks. Each half cycle of rotation contained an acceleration phase followed by a deceleration phase, and the late half-cycle of rotation always sped up to approximately 5–7 m/s. The mean (± SEM) of the maximal velocity was 5.9 ± 0.6 m/s (n = 6). The rotational velocity usually peaked between 105°and285°.

### Function of abdominal rotation in a free pupa

**Figure 7.**
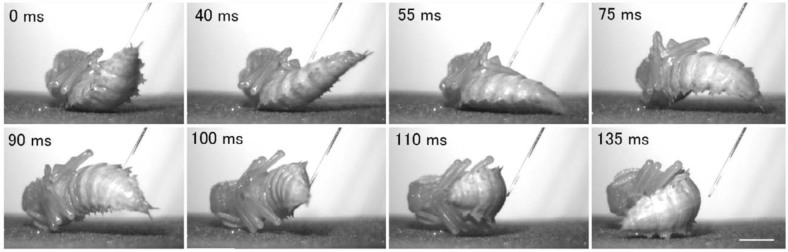
Turning of the pupal body driven by the abdominal rotation response in a pupa lying dorsal side down on flat sandpaper. The time in each frame indicates the time elapsed from the start of body movement. Scale bar = 5 mm. High quality figures are available online.

**Figure 8.**
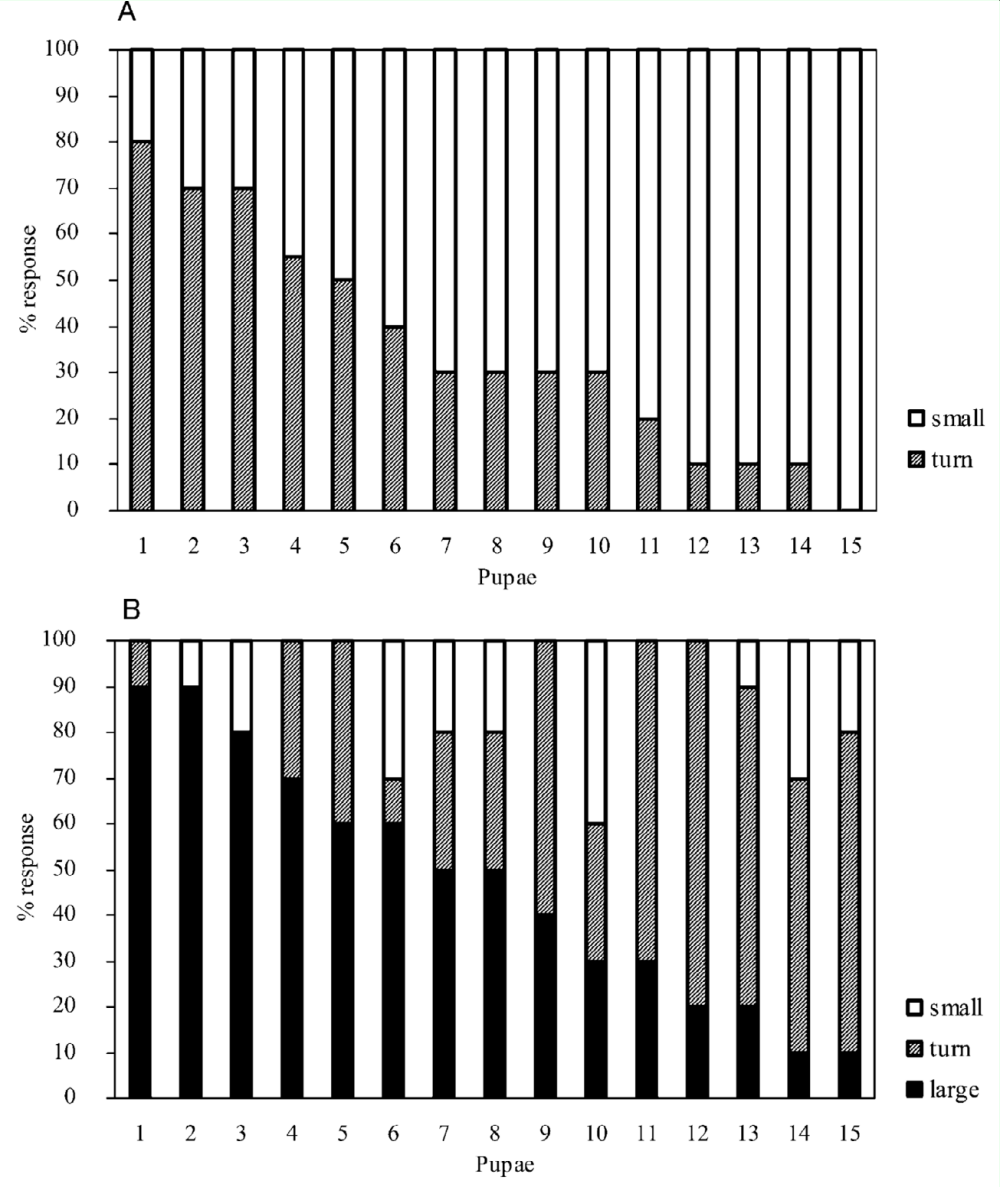
Rates of body turning or displacement induced by abdominal rotation responses in freely lying pupae. Pupae were laid dorsal side down (A) or lateral side down (B) on a rough substrate. Turn was defined as a 90° or 180° turn of the pupal body around the longitudinal axis of the body. Small and large movements were defined as a < 5 mm and > 5 mm displacement of the center of pupal body from its initial position, respectively. Different groups of pupae were used in the two experiments. High quality figures are available online.

To elucidate the behavior of pupae under a free, unrestrained condition, pupae were laid dorsal or lateral side down on a rough substrate. When a pupa lying dorsal side down was stimulated, the abdominal rotation response often made the pupa turn its dorsum toward the source of stimulation. Furthermore, analyses of sequential frames of high-speed photography revealed that a pair of thorn-like processes (urogomphi) extending from the tip of the abdomen served as a fulcrum for transmitting the power of abdominal rotation to the 90° turn of the pupal body around the longitudinal axis of the body (Figure 7). When the abdomen bent backward strongly (panel 75 ms, Figure 7), the urogomphi pressed the substrate obliquely, and the reaction force from the substrate and the momentum produced by the rapid flexion of the abdomen during the late half cycle of rotation appeared to drive the turning of the pupal body (panels 90-135 ms, Figure 7). When abdominal rotation failed to turn the pupal body mainly due to slippage of the urogomphi, it usually caused a small displacement of the pupal body (Figure 8A). When a pupa lying lateral side down was stimulated, an abdominal rotation response usually induced a 180° turn of the pupal body or a relatively large movement of the pupal body away from the source of stimulation (Figure 8B). In such cases, the turning or movement was performed with the aid of the urogomphi during the late half cycle of rotation (data not shown).

Since *Z. atratus* pupae are usually hidden within a small chamber (pupal cell) made in the soil under the natural environment, whether the pupae could perform such defensive movements inside the pupal cell was examined. Pupae placed in the center of an artificial pupal cell usually could make a complete turn without touching the wall of pupal cell with its urogomphi; however, turning was restricted when the pupa was placed near the wall. Thus, the defensive turn and displacement of the pupal body may be an effective defensive mechanism in its natural habitat.

## Discussion

The exposed appendages of the exarate pupae of *Z. atratus* appear to be the most vulnerable parts of its body and are easily attacked by relatively larger predators, including cannibalistic larvae of the same species. The pupal abdominal rotation response functions as an effective defense against such enemies (Ichikawa and Kurauchi 2009). How can this response have an anti-predator function? Since each abdominal segment is armed with sclerotized spines or jaws, vigorous rotation of the abdominal segments themselves may be effective for startling or deterring some predators. Furthermore, the rotation response can change the orientation or spatial position of the pupal body, and this change leads the pupa to move away from or avoid the enemy as observed in *T. molitor* (Askew and Kurtz 1974). In the present study, a similar abdominal rotation in a free *Z. atratus* pupa lying down on a substrate was observed. The abdominal rotation often made the pupa turn its dorsum toward the source of stimulation with the aid of the urogomphi, which served as a fulcrum (Figures 7 and 8). The dorsum has a relatively flat and hardened cuticle and may function as a shield protecting the pupa from predators. The shield is not a senseless guard; it may be able to monitor the action of the potential enemy using the many mechanosensory hairs and campaniform sensilla present on almost all parts of the shield (Kurauchi et al. 2011).

The pupal abdominal rotation is a stereotypical response occurring in an all-or-none fashion, although the amplitude of the horizontal component of the rotation is often variable within a (small) range, depending on the intensity of stimulation (i.e., the number of sensilla stimulated) (Figure 2) and the site of stimulation (Figure 4). The abdomen rotated in different directions depending on the side of stimulation (Figure 3). Simultaneous stimulation of appendages on both sides often induced variable and complex patterns of abdominal movements (Figure 7). These findings suggest that the central nervous system may have two neuronal mechanisms that generate motor patterns that rotate the abdomen in opposite directions. In a preliminary experiment, a transection of the ventral nerve cord between the mesothoracic and metathoracic ganglia failed to block the abdominal rotation in response to a tactile stimulation of the hind leg, although the sensitivity to the stimulation was greatly reduced. Thus, such a pattern generation mechanism (or a pattern generator) may be located in the abdominal ganglia. Since the sensory axons originating from mechanoreceptors on the cephalic and thoracic appendages usually terminate in the brain-subesophageal ganglion complex and thoracic ganglia, respectively (Breidbach 1990), the sensory information from receptors may be transferred to interneurons that descend the ventral nerve cord to activate the pattern generators. The complex trajectory patterns of abdominal rotations induced by the bilateral stimulation may indicate an interaction or interference between the two pattern generators producing opposite abdominal rotations. The simultaneous stimulation of appendages on both sides may not always indicate the simultaneous activation of two pattern generators, because the latency of a rotational response is variable. It is possible that when either of the two generators is activated at an earlier time, the early generator may dominate to determine abdominal rotation patterns like those illustrated in Figure 7C–F. If two generators are activated synchronously, the abdominal movement pattern may become the sum of the clockwise and anticlockwise rotation patterns, such as the pattern shown in Figure 7G. The relatively longer latency appears to be a temporal characteristic of rotational responses induced by the bilateral stimulations (Figure 7). Since simultaneous stimulation of bilateral appendages may be very unlikely under natural conditions, the results possibly indicate some confusion in the sensory signal processing mechanism. The putative two-generator model may be able to account for most of the results qualitatively. However, the model requires some modification to account for the quantitative aspects of the results (see Ichikawa et al. 2012), because the trajectory patterns revealed by stimulation of different appendages sometimes differed significantly (Figure 4A).

In general, a neural (or central) pattern generator is postulated to produce a cyclic motor pattern such as walking, flying, swimming, or breathing (Delcomyn 1980; Marder and Buchner 2001). The pupal pattern generator postulated in the present study may be regarded as a simple pattern generator that produces only one motor pattern cycle. In general, a simple circular (or elliptical) pattern known as a Lissajous figure can be easily visualized on an oscilloscope by applying two sine waves with a phase difference of 90° (or < 90°) to the horizontal and vertical deflecting plates of the oscilloscope. If an ideal abdominal segment has two sets of antagonistic muscles that are functionally equivalent to the two sets of electrostatic deflecting plates, and the two are supplied with different sinusoidal motor signals from the putative pattern generator, the segment may be able to perform a circular rotation. The pupae of *Z. atratus* have nine abdominal segments (numbered A1—A9) that are slightly compressed in the dorsoventral axis and have an almost elliptical cross section (Kurauchi et al. 2011). Each abdominal segment of A2—A6 has four longitudinal (intersegmental) muscle bundles that are located on the four sides of a rhombus inscribed in the elliptical segment (T. Ichikawa, unpublished observation); the muscular arrangement in the segments may almost fulfill the structural requirements for generating the circular motion. However, the temporal pattern of the putative motor signals may not be a simple sinusoidal pattern, because the speed of abdominal rotation changes substantially during the course of rotation (Figure 6). The strong bending of the abdomen during the abdominal rotation (Figure 1B) suggests that the intersegmental muscles in all segments may participate in abdominal rotation and that analogous muscles in different segments may be activated in (near) synchrony during an appropriate phase of the rotation. Electrophysiological studies will reveal the central motor pattern and muscle activation patterns during the pupal abdominal rotation.
